# COL4A1 promotes the growth and metastasis of hepatocellular carcinoma cells by activating FAK-Src signaling

**DOI:** 10.1186/s13046-020-01650-7

**Published:** 2020-08-03

**Authors:** Ting Wang, Haojie Jin, Jingying Hu, Xi Li, Haoyu Ruan, Huili Xu, Lin Wei, Weihua Dong, Fei Teng, Jianren Gu, Wenxin Qin, Xiaoying Luo, Yujun Hao

**Affiliations:** 1 Shanghai Medical College of Fudan University, Shanghai, 200032 People’s Republic of China; 2 State Key Laboratory of Oncogenes and Related Genes, Shanghai Cancer Institute, Renji Hospital, Shanghai Jiao Tong University School of Medicine, Shanghai, 200240 People’s Republic of China; 3 The Fifth Affiliated Hospital of Sun Yat-sen University, Zhuhai, Guangdong 519000 People’s Republic of China; 4 Changzheng Hospital, Navy Medical University, Shanghai, 200003 People’s Republic of China

**Keywords:** COL4A1, HCC, RUNX1, FAK, Src

## Abstract

**Background:**

Collagens are the most abundant proteins in extra cellular matrix and important components of tumor microenvironment. Recent studies have showed that aberrant expression of collagens can influence tumor cell behaviors. However, their roles in hepatocellular carcinoma (HCC) are poorly understood.

**Methods:**

In this study, we screened all 44 collagen members in HCC using whole transcriptome sequencing data from the public datasets, and collagen type IV alpha1 chain (COL4A1) was identified as most significantly differential expressed gene. Expression of COL4A1 was detected in HCC samples by quantitative real-time polymerase chain reaction (qRT-PCR), western blot and immunohistochemistry (IHC). Finally, functions and potential mechanisms of COL4A1 were explored in HCC progression.

**Results:**

COL4A1 is the most significantly overexpressed collagen gene in HCC. Upregulation of COL4A1 facilitates the proliferation, migration and invasion of HCC cells through FAK-Src signaling. Expression of COL4A1 is upregulated by RUNX1 in HCC. HCC cells with high COL4A1 expression are sensitive to the treatment with FAK or Src inhibitor.

**Conclusion:**

COL4A1 facilitates growth and metastasis in HCC via activation of FAK-Src signaling. High level of COL4A1 may be a potential biomarker for diagnosis and treatment with FAK or Src inhibitor for HCC.

## Background

Liver cancer is one of most malignant cancer and causes more than 700,000 deaths per year worldwide [1]. Hepatocellular carcinoma (HCC) is the most common type of liver cancer, accounting for approximately 90% of all liver cancers [2]. Although surgical resection, orthotopic liver transplantation, radiofrequency thermal ablation and targeted therapy have been applied clinically to liver cancer treatment, the 5-year overall survival rate is still low due to late diagnosis, disease recurrence, metastasis, and the complicated etiology of HCC [3]. Therefore, it is necessary to further understand the molecular mechanisms and identify novel targets for diagnosis as well as treatment of HCC.

Collagens are the most abundant proteins in the human body, accounting for one third of total proteins. In humans, there are at least 28 different types of collagen proteins encoded by 44 collagen genes [4]. They are essential in extra cellular matrix (ECM) which is the major component in tumor microenvironment and can regulate tumor cell behaviors [5, 6]. Collagen proteins are produced and secreted by fibroblasts [7], osteoblasts [8], hematopoietic cells [9], and also can be biosynthesized and regulated by endothelial cells [10, 11] and cancer cells [12]. Over the last few years, accumulated evidences have indicated that some collagens are differentially expressed in cancer. Collagen type I contributes to pancreatic, lung, bladder, liver and breast cancer progression [13–17]. Collagen type IV (Col IV) levels are elevated in patients with colorectal liver metastases [18, 19]. Collagen type VI has been identified as biomarker of prognosis for colorectal, ovarian, and pancreatic cancer [20–22]. Collagen type XI is highly expressed in breast cancer, colorectal cancer, and metastatic ovarian carcinoma [23–25]. Collagen type XVII is overexpressed in skin cancers, such as squamous cell carcinoma and melanoma [26, 27]. However, the functions and mechanisms of collagen genes in HCC are still largely unknown.

Collagen IV is the most abundant constituents of basement membranes of ECM [28]. Col IV is majorly generated and secreted from fibroblasts, endothelial cells, epithelial cells, and some tissue specific cells (e.g. hepatic stellate cells) [29, 30]. COL4A1 and COL4A2 encode Collagen IV alpha 1 chain and alpha 2 chain, and assemble into α1α1α2 heterotrimers (Col IV) then secreted into extracellular matrix [31]. COL4A1 mutations have been identified in vascular abnormalities, myopathy, nephropathy, and Walker-Warburg syndrome [32–34]. Upregulated COL4A1 promotes tumor invasion via induction of tumor budding in bladder cancer cells [35]. Overexpressed COL4A1 contributes to the proliferation and migration of breast cancer cells [36]. However, the detailed mechanisms of COL4A1 in HCC progression has not been elucidated.

In this study, expression profiles of collagen genes were comprehensively screened and COL4A1 was identified as the most abundant and significantly differential expressed collagen gene in HCC. Upregulation of COL4A1 promoted the proliferation and metastasis of HCC cells through FAK-Src signaling. We first found that expression of COL4A1 is upregulated by transcriptional factor RUNX1. Moreover, we innovatively discovered that HCC cells with high COL4A1 expression were sensitive to the treatment of FAK or Src inhibitor. Our findings suggest that the upregulation of COL4A1 is important in the progress of HCC, and COL4A1 maybe a potential target for diagnosis and treatment of HCC.

## Materials and methods

### Clinical specimens and TCGA data analysis

89 pairs of HCC tissues and peritumor samples for quantitative real-time polymerase chain reaction (qRT-PCR) analyses, 10 pairs of HCC tissues and peritumor samples for western blot analyses and 4 pairs of paraffin-embedded tissue for immunohistochemistry (IHC) analyses were obtained from patients at Zhongshan Hospital of Fudan University (Shanghai, China) from 2004 to 2005. Ethical approval was examined and certified by the Ethics Committee of Zhongshan Hospital Biomedical Research Department, and written informed consent was obtained from all involved patients. The slides of paraffin-embedded HCC tissues and paired peritumor samples for IHC analyses were purchased from Liaoding (Shanghai, China) (*n* = 20). The whole transcriptome sequencing (RNA-seq) data of 374 liver tumor tissues and 50 adjacent non-tumor tissues were obtained from The Cancer Genome Atlas (TCGA) liver cancer dataset (LIHC) (http://cancergenome.nih.gov). mRNA level of COL4A1 of 20 tumor types and another 4 datasets of HCC were obtained from the Oncomine database (https://www.oncomine.org/resource/main.html). Representative IHC staining results of COL4A1 in HCC and normal liver tissues were obtained from the Human Protein Atlas online database (https://www.proteinatlas.org, magnification, × 40).

### Cell lines and culture

Human HCC cell lines (HepG2, PLC/PRF/5, Hep3B, and SK-Hep1) were purchased from the American Type Culture Collection (Manassas, VA, USA). HCC cell line (Huh7) was provided by Riken Cell Bank (Tsukuba, Japan). Human normal liver cell line (L02) and HCC cell line (SMMC7721) were purchased from Cell Bank of the Chinese Academy of Sciences (Shanghai, China). All cell lines were cultured in Dulbecco’s modified Eagle’s medium (DMEM; Gibco, USA) supplemented with 10% fetal bovine serum (FBS; Gibco, USA), 100 units/ml penicillin and 100 μg/ml streptomycin (Gibco, USA), and incubated at 37 °C in a humidified incubator containing 5% CO2.

### Antibodies and plasmids

Antibodies used in this study were shown in Additional file 1: Table S1. Human expression vectors for HA-RUNX1, Myc-RUNX2 and Myc-RUNX3 recombinant proteins were generated using pCMV backbone vector. The promoter of COL4A1 was cloned into pGL3 basic vector. The subcloning primers were listed in Additional file 2: Table S2.

### Western blot

Cells were washed three times with cold phosphate buffered saline (PBS) and total cellular protein was extracted using RIPA lysis buffer (Qiagen, Germany) supplied with proteinase inhibitor cocktail and phosphatase inhibitor (Roche Applied Science, Switzerland). The lysates were incubated on ice for 30 min followed by centrifugation at 4 °C 12000×g for 30 min. Protein concentrations were analyzed using the Bicinchoninic Acid (BCA) Kit (Pierce, Rockford, IL). 40 μg of total proteins were separated by 10% sodium dodecyl sulfate-polyacrylamide gel electrophoresis (SDS-PAGE) and transferred onto 0.22 μm polyvinylidene fluoride membrane (PDVF; Millipore). The membranes were blocked with 5% non-fat dried milk for an hour at room temperature, and then incubated with primary antibodies overnight at 4 °C. Protein bands were visualized by the enhanced chemiluminescence (ECL) detection kit (Tanon, China). Quantification of western blots were analyzed by Image J.

### Immunohistochemistry (IHC)

Paraffin-embedded HCC tissues and peritumor samples were completely deparaffinized, and then performed antigen retrieval using antigen retrieval solution (Beyotime, Shanghai, China). Immunohistochemistry staining were applied with Immunohistochemistry Application Solutions Kit (Cell Signaling Technology, Dancers, USA) according to the manufacturer’s instructions. Briefly, the slides were incubated with primary anti-COL4A1 (1:200, Abcam) overnight at 4 °C and incubated the secondary antibody 30 min at room temperature. Then, the slides were counter stained with hematoxylin for 3 min. Staining results were independently evaluated by two experienced pathologists who were blinded to all clinical data.

### RNA extraction and qRT-PCR

Total RNA was extracted from cell lines and tissue samples using the TRIzol kit (Invitrogen, Carlsbad, CA, USA). RNA (1 μg) was reverse-transcribed into cDNA immediately using Prime-Script RT kit (Takara, Shiga, Japan) following manufacturer’s instructions. qRT-PCR was carried out with SYBR Premix EX Tag (Takara) on an ABI Prism 7500 fast RT-PCR instrument (Applied Biosystems, Foster City, CA). Each experiment was performed in triplicate. β-actin was used as the internal reference gene. Data were acquired during the extension step. Objective CT values were normalized to β-actin and 2^-△Ct^ method was used to calculate relative mRNA levels of gene expression. Primer sequences for qRT-PCR were listed in Additional file 2: Table S2. The qRT-PCR primers for COL4A1 are not exon spanning type, but their specificity has been tested.

### Lentiviral constructs and cell infection

To knockdown COL4A1 in cell lines, two independent shRNA sequences were designed and cloned into the pGreen-Puro vector (System Biosciences, CA). Another shRNA with a non-targeting sequence was used as a negative control (NC). The shRNA sequences were listed in Additional file 2: Table S2. Virus packaging was performed in HEK 293 T cells after co-transfection of pGreenPuro-shCOL4A1 with packaging plasmid pPACK-GAG, pPACK-REV (System Biosciences) and envelope plasmid pVSV-G (System Biosciences) using Lipofectamine 3000 (Invitrogen, Carlsbad, CA, USA). Viruses were harvested 48 h after transfection. Medium with viral supernatant was filtered through a 0.45 μm strainer and viral titers were determined. SMMC7721 cells and SK-Hep1 cells were infected with lentivirus using polybrene (6 μg/ml, Sigma).

CRISPR/Cas9 Synergistic Activation Mediator (SAM) is an engineered protein complex, which is a powerful tool for strong transcriptional activation of endogenous genes at targeted sites. Complete SAM system consists of two separate lentiviral vectors: the dCas9-VP64-puro vector and the sgRNA (COL4A1)-MS2-P65-HSF1-G418 (single guiding RNA) vector. Plasmids design and two lentiviruses packaging were done at Genechem Co., Ltd. (Shanghai, China). sgRNAs with matching COL4A1 gene promoter sequences (Gene Bank ID: NM_001845) were listed in Additional file 2: Table S2. HepG2 cells and PLC/PRF/5 cells were infected with the lentivirus of dCas9-VP64-puro. After two weeks of puromycin (1 μg/ml) selection, cells were infected with indicated sgRNA (COL4A1)-MS2-P65-HSF1-G418 lentivirus and selected with G418 (600 μg/ml).

### siRNA knockdown

The siRNAs targeting human COL4A2, COL3A1, COL1A1, RUNX1 and the scramble siRNA control were obtained from Biotend (Shanghai, China). siRNAs were transfected into indicated cells with Lipofectamine 3000 according to manufacturer’s instructions. Cells were harvested 48–72 h post transfection for various assays. Sequences for siRNA were listed in Additional file 2: Table S2.

### Proliferation, migration and invasion analysis

Cell proliferation, invasion and migration assays were measured with the xCELLigence System’s Real-Time Cell Analyzer (RTCA, Roche/ACEA Biosciences) placed in a humidified incubator and maintained at 37 °C with 95% air/5% CO2. This system continuously monitored electrical impedance which created by cell adhesion and proliferation in microelectrode-integrated membrane, and outputted as a unit-less parameter (cell index). For proliferation assays, 1× 10^4^ to 3× 10^4^ cells were seeded into E-plate 16 (ACEA Biosciences) with 200 μL DMEM containing 10% FBS (*n* = 3). Cell index was normalized to baseline reading at time point 0, and measured every 30 min for 72 h. Migration and invasion assays were performed in 16-well CIM plates (ACEA Biosciences). For migration assays, 1.5× 10^5^ cells were seeded as triplicates in the upper chamber in serum free medium. Upper chamber was then placed on the lower part of the CIM-device containing DMEM with 10% FBS as a chemoattractant. Cell index was measured every 30 min for 48 h. For invasion assays, upper chamber of CIM-16 plate was initially coated with Matrigel (BD Biosciences, Bedford, MA, USA) diluted in serum free medium at a ratio of 1:20. Then, next steps were same with migration assays.

For cell proliferation assay, Cell Counting Kit-8 (Dojindo, Japan) was also applied according to the manufacturer’s instructions. Briefly, 3× 10^3^ cells per well were planted in a 96-well plate. Absorbance at OD450 was measured for 4 consecutive days and used to plot cell growth curves. For colony formation assay, 8× 10^2^ cells were seeded in 6-well plates and maintained in DMEM medium with 5% FBS. After 14 days, cells were washed with PBS and stained with 0.5% crystal violet. For transwell assay, 5× 10^4^ cells were seeded into upper chambers (transwells with 8-μm pores, Corning, USA) without FBS, and DMEM containing 10% FBS was introduced to the lower chambers (24-well plates). After 2 days, cells remaining on the upper surface of the filter were removed using a cotton swab with PBS, then transwells were fixed and stained with 0.5% crystal violet. The migratory cells were photographed and counted in 5 different fields per well.

### Drug treatment

FAK inhibitor Defactinib (VS-6063) was purchased from CSNpharm (CSN17445, Shanghai, China). Src inhibitor Saracatinib (AZD0530) was purchased from Selleck Chemical (S1006, Huston, TX, USA). To evaluate inhibitory activity of FAK or Src inhibitor (Defactinib or Saracatinib), cells were firstly seeded at a density of 5× 10^3^ in 96-well plates and incubated overnight. Then Defactinib or Saracatinib was added at indicated concentrations. After 2 days, CCK8 was applied to measure survival cells following manufacturer instructions.

### Wound healing assay

Cells were grown in 12-well plates at 95% confluency. A linear wound was scratched with a 200 μL sterile pipette tip across the monolayers. After washing with PBS to remove cell debris, adherent cells were incubated in medium with 10% FBS. Wounded monolayers were photographed every 3 h for 24 h.

### Tumor xenograft models

Subcutaneous xenograft mouse model was used to assess tumor growth. Animal experiments were approved by the Ethics Committee of the Renji Hospital, Shanghai Jiao Tong University School of Medicine. Female nude mice (age, 4–5 weeks; weight, 14–16 g; Institute of Zoology, Chinese Academy of Sciences) were randomly divided into three groups: two COL4A1 knockdown groups and one NC group (*n* = 7 per group). A total of 2 × 10^6^ SMMC7721 cells in 100 μL of DMEM without FBS were injected into right axillary fossa of nude mice. Tumor volume was measured by caliper measurements every 3 days and calculated with the formula of (length × width^^2^)/2.

### Statistical analysis

Data were analyzed using GraphPad Prism 7. Results were presented as mean ± standard deviation (SD, *n* = 3). Statistical differences between groups were evaluated by the student’s *t* test (paired/unpaired). Pearson correlation tests were performed on correlation analyses. Two-way analysis of variance (ANOVA) followed by Tukey’s multiple comparisons test was performed to compare significant difference and calculate the *P*-value between the different groups. It was considered as statistically different when *P* < 0.05 (*P < 0.05, **P < 0.01, ***P < 0.001, ****P < 0.0001), otherwise not significant (ns).

## Results

### COL4A1 is upregulated in HCC

**Fig. 1:**
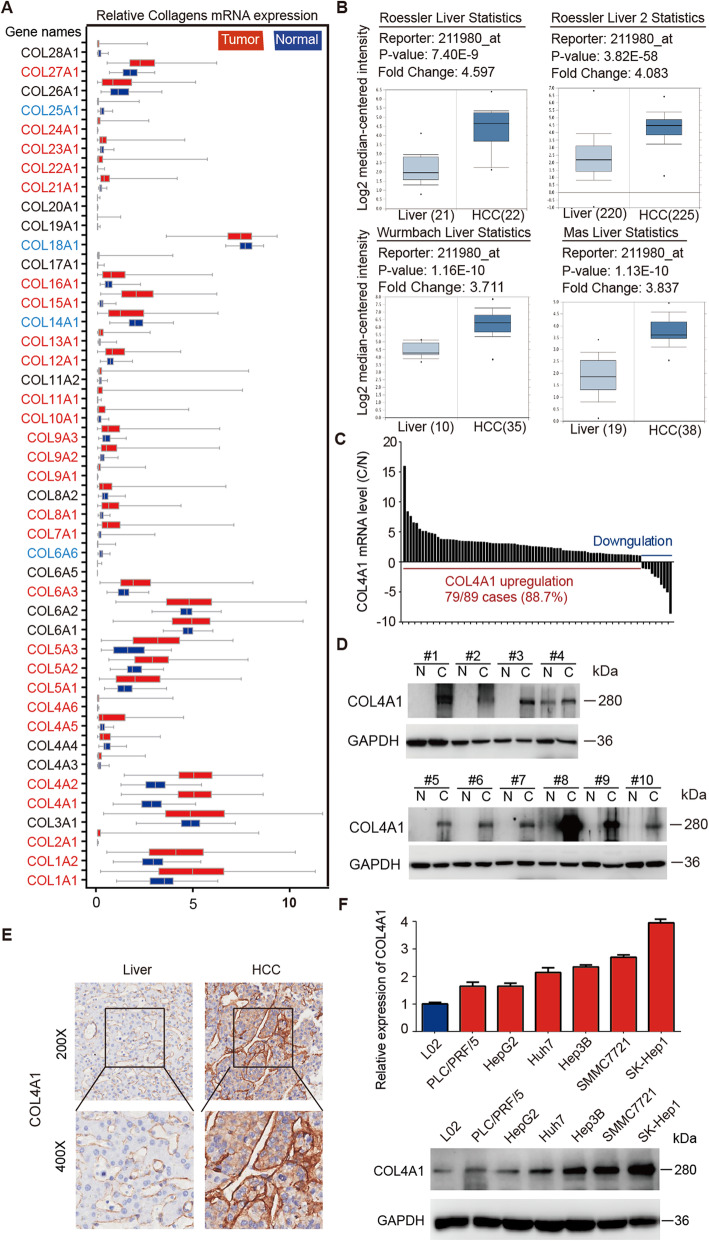
Upregulation of COL4A1 in HCC. **a** Box**-**whisker Plot indicated the mRNA expression profiles of 44 collagen genes in TCGA dataset. The gene name shown in red and blue illustrates the 27 upregulation genes and 4 downregulation genes in tumor tissues compared with normal liver tissues, respectively. **b** Expression of COL4A1 was significantly upregulated in HCC compared with normal liver tissues in 4 datasets, including Roessler Liver Statistics, Roessler Liver 2 Statistics, Wurmbach Liver Statistics, and Mas Liver Statistics. **c** mRNA levels of COL4A1 in paired HCC samples were detected by qRT-PCR (*n* = 89). Fold changes (C/N) were presented. C, cancerous tissues; N, noncancerous liver tissues. **d** The protein levels of COL4A1 in 10 paired cancerous tissues (C) and the matched adjacent noncancerous liver tissues (N) from HCC patients were analyzed by western blot. **e** COL4A1 proteins were highly expressed in HCC tissues. Immunohistochemistry staining of COL4A1 was performed in paired HCC samples and normal liver tissues (*n* = 24). The representative images were shown. **f** Expression levels of COL4A1 were measured by qRT-PCR and western blot in indicated HCC cell lines

To identify the cancer-related collagen genes in HCC, we first analyzed expression level for all 44 members of collagen genes in HCC using RNA-seq data from TCGA-LIHC dataset. 31 of 44 (70.5%) collagen genes were differentially expressed in 374 liver cancer samples compared with 50 normal liver samples. Among 27 upregulated genes and 4 downregulated genes, COL1A1, COL1A2, COL4A1, and COL4A2 were significantly upregulated genes (Fig. 1a and Additional file 3: Table S3). In 50 paired HCC and normal liver samples from TCGA dataset, COL1A1 and COL1A2 were only upregulated in 74% (37 of 50) of HCC samples, but COL4A1 and COL4A2 were upregulated in 100% (50 of 50) of HCC samples (Additional file 4: Figure S1A). Furthermore, COL4A1 was the most significantly upregulated collagen gene in HCC in term of average fold changes (cancerous tissues/noncancerous liver tissues, 1.69 for COL1A1; 1.69 for COL1A2; 2.10 for COL4A1 and 1.92 for COL4A2). This result was confirmed by other four HCC datasets (Roessler Liver Statistics, Roessler Liver 2 Statistics, Wurmbach Liver Statistics, and Mas Liver Statistics) in Oncomine database (Fig. 1b). This result was also validated in our own dataset which we submitted previously (GSE84402) [37], and COL4A1 was upregulated in 11 of 14 HCC samples (Additional file 4: Figure S1B). In another 89 pairs of HCC and adjacent normal tissues, COL4A1 was upregulated in 79 of 89 HCC samples (88.7%) by qRT-PCR analysis (Fig. 1c and Additional file 4: Figure S1C). In addition, COL4A1 was upregulated not only in HCC but also in most types of cancer, including colorectal cancer, gastric cancer as well as head and neck cancer, etc. (Additional file 4: Figure S1D).

Since mRNA levels of genes are not always consistent with their protein levels, protein level of COL4A1 in clinical HCC tissues needs to be further analyzed. Firstly, COL4A1 was highly expressed in all HCC samples (10 of 10) compared with normal liver tissues by western blot analyses (Fig. 1d). Secondly, we performed IHC staining of COL4A1 in clinical specimens, and COL4A1 was highly expressed in 17 of 24 (70.8%) human HCC specimens compared with normal liver tissues (Fig. 1e). We also analyzed the data of IHC staining in the Human Protein Atlas database, consistently, moderate COL4A1 staining was observed in HCC samples and no obvious staining of COL4A1 was found in hepatocytes (Additional file 4: Figure S1E).

We next examined the mRNA and protein levels of COL4A1 in six HCC cell lines (HepG2, Hep3B, SK-Hep1, SMMC7721, Huh7, and PLC/PRF/5) and an immortalized liver cell line L02. As shown in Fig. 1f, both mRNA and protein levels of COL4A1 were higher in HCC cell lines than that in L02. Since Col IV is normally secreted to extracellular matrix to perform its function [38], we detected the secretion of COL4A1 in medium supernatant of HCC cell lines. As shown in Additional file 4: Figure S1F, more COL4A1 was secreted into culture medium of SK-Hep1 and SMMC7721 cells which expressed high levels of COL4A1, but low levels of COL4A1 in culture medium of HepG2 and PLC/PRF/5 cells which expressed relative low levels of COL4A1, indicating that cells with high expression of COL4A1 will secrete more COL4A1 into the extracellular matrix.

COL4A1 and COL4A2 are only 127 bp apart on chromosome 13q34 in a head-to-head arrangement and share a bidirectional promoter. COL4A2 expression is significantly positively correlated with COL4A1 [31, 39]. COL1A1 has been reported to overexpress in HCC samples [40]. But the expression of COL3A1 is similar in HCC samples compared with non-tumor liver tissues (Fig. 1a). So, we checked the mRNA and protein levels of COL4A2, COL1A1 and COL3A1 in HCC cell lines as positive or negative controls. As shown in Additional file 4: Figure S1G&H, both mRNA and protein levels of COL4A2 were higher in HCC cell lines than that in L02, which was consistent with the trend of COL4A1. Although COL1A1 was highly expressed in HCC samples (Fig. 1a), the expression of COL1A1 in HCC cell lines was not more than L02 cells (Additional file 4: Figure S1G&H), suggesting that other cell types in tumor microenvironment might contribute more for COL1A1 expression than HCC cells. The expression of COL3A1 had no difference in HCC cell lines compared with L02 (Additional file 4: Figure S1G&H).

We analyzed correlation between COL4A1 expression and clinicopathological parameters of HCC. Although not obvious, statistically, the expression of COL4A1 were slightly higher in higher stages of HCC (III and IV) than that in lower stages (I and II) (Additional file 4: Figure S1I). Together, our results indicate that COL4A1 is most significantly upregulated collagen gene in HCC cells and may plays critical roles in HCC progression.

### COL4A1 promotes proliferation, migration and invasion of HCC cells

**Fig. 2:**
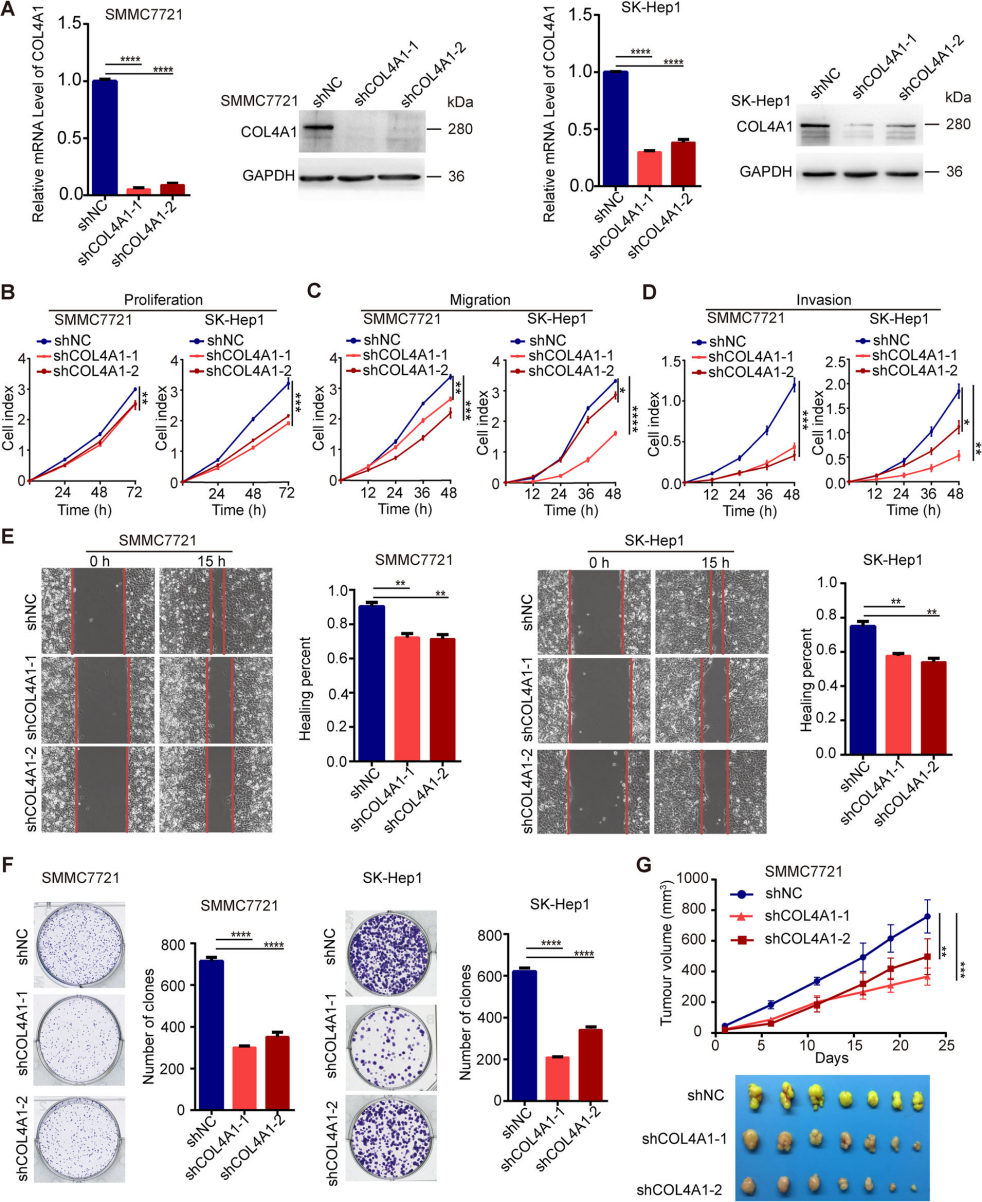
COL4A1 knockdown inhibits the proliferation, migration and invasion of HCC cells. **a** Knockdown efficiency of COL4A1 using shRNA was confirmed by qRT-PCR and western blot in SMMC7721 cells and SK-Hep1 cells. **b-d** COL4A1 knockdown inhibited cell proliferation (**b**), migration (**c**) and invasion(**d**) by Real-time cell analyzer. **e** Downregulation of COL4A1 inhibited cell migration by wound healing assay. **f** COL4A1 knockdown inhibited cell proliferation by colony formation assay. **g** COL4A1 knockdown suppressed tumor growth in vivo. *n* = 7/group. Tumor volume was measured, and photographs of tumors were taken. Data are presented as means ± standard deviation. Student *t* test and Two-way ANOVA followed by Tukey’s multiple comparisons test, *P < 0.05, **P < 0.01, ***P < 0.001, ****P < 0.0001

According to the expression patterns of COL4A1 in HCC cell lines, we knocked down COL4A1 in SMMC7721, SK-Hep1, HepG2 and PLC/PRF/5 cells by two independent shRNAs (Fig. 2a and Additional file 5: Figure S2A). Knockdown of COL4A1 significantly inhibited the proliferation (Fig. 2b and Additional file 5: Figure S2B), migration (Fig. 2c and Additional file 5: Figure S2C) and invasion (Fig. 2d and Additional file 5: Figure S2D) in those cells by real-time cell analyzer (RTCA). These data were validated by wound healing assay and colony formation assay (Fig. 2e & f). Moreover, we conducted subcutaneous xenograft tumor experiments in nude mice, and results showed that knockdown of COL4A1 significantly reduced the growth of subcutaneous xenograft tumors derived from SMMC7721 cells (Fig. 2g).

**Fig. 3:**
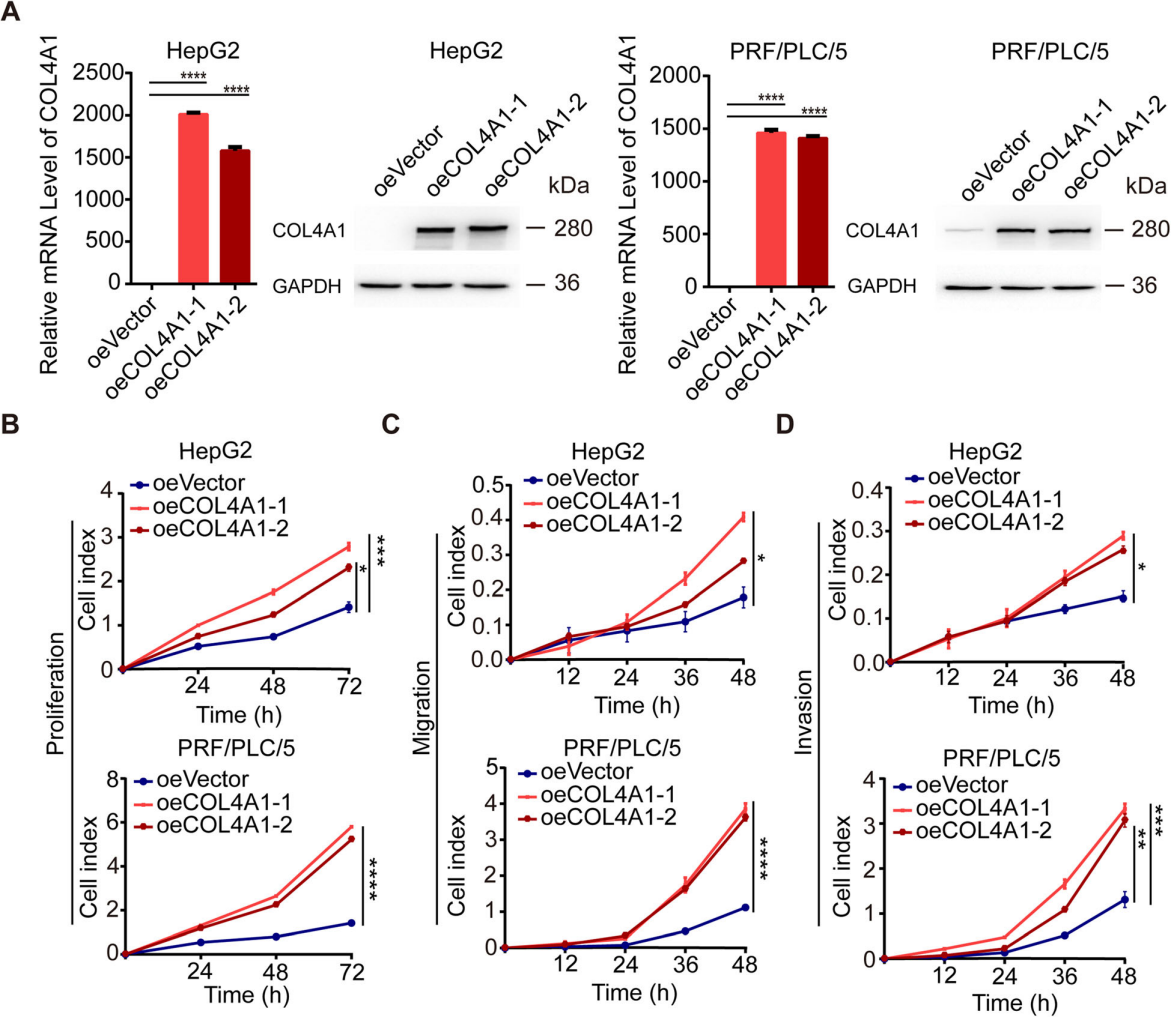
COL4A1 overexpression promotes the abilities of cell proliferation, migration and invasion in HCC cells. **a** CRISPR/Cas9/Synergistic Activation Mediator (SAM) - mediated overexpression of COL4A1 in HepG2 cells and PLC/PRF/5 cells were confirmed by qRT-PCR and western blot. **b-d** Stable overexpression of COL4A1 promoted cell proliferation (**b**), migration (**c**) and invasion (**d**). Data are presented as means ± standard deviation. Student *t* test and Two-way ANOVA followed by Tukey’s multiple comparisons test, *P < 0.05, **P < 0.01, ***P < 0.001, ****P < 0.0001

We next overexpressed COL4A1 in HepG2 cells and PLC cells by CRISPR/Cas9 synergistic activation mediator (SAM) strategy (Fig. 3a). Overexpression of COL4A1 promoted the abilities of cell proliferation, migration and invasion (Fig. 3b-d).

Given that COL4A1 normally perform its function through assembling as heterotrimers with COL4A2, we explored whether COL4A2 would have similar biological function as COL4A1. As shown in Additional file 6: Figure S3, knockdown of COL4A2 in SMM7721 and SK-Hep1 by siRNA could inhibit the proliferation and migration of those cells. In addition, COL1A1 has been shown to promote HCC progression [17, 40, 41]. We also found that knockdown of COL1A1 inhibited the proliferation and migration of SMM7721 and SK-Hep1 cells (Additional file 7: Figure S4). However, knockdown of COL3A1 had no impact on HCC cells proliferation and migration (Additional file 8: Figure S5). Taken together, collagen IV in liver cancer cells could promote HCC progression.

### COL4A1 is transcriptionally activated by RUNX1

**Fig. 4:**
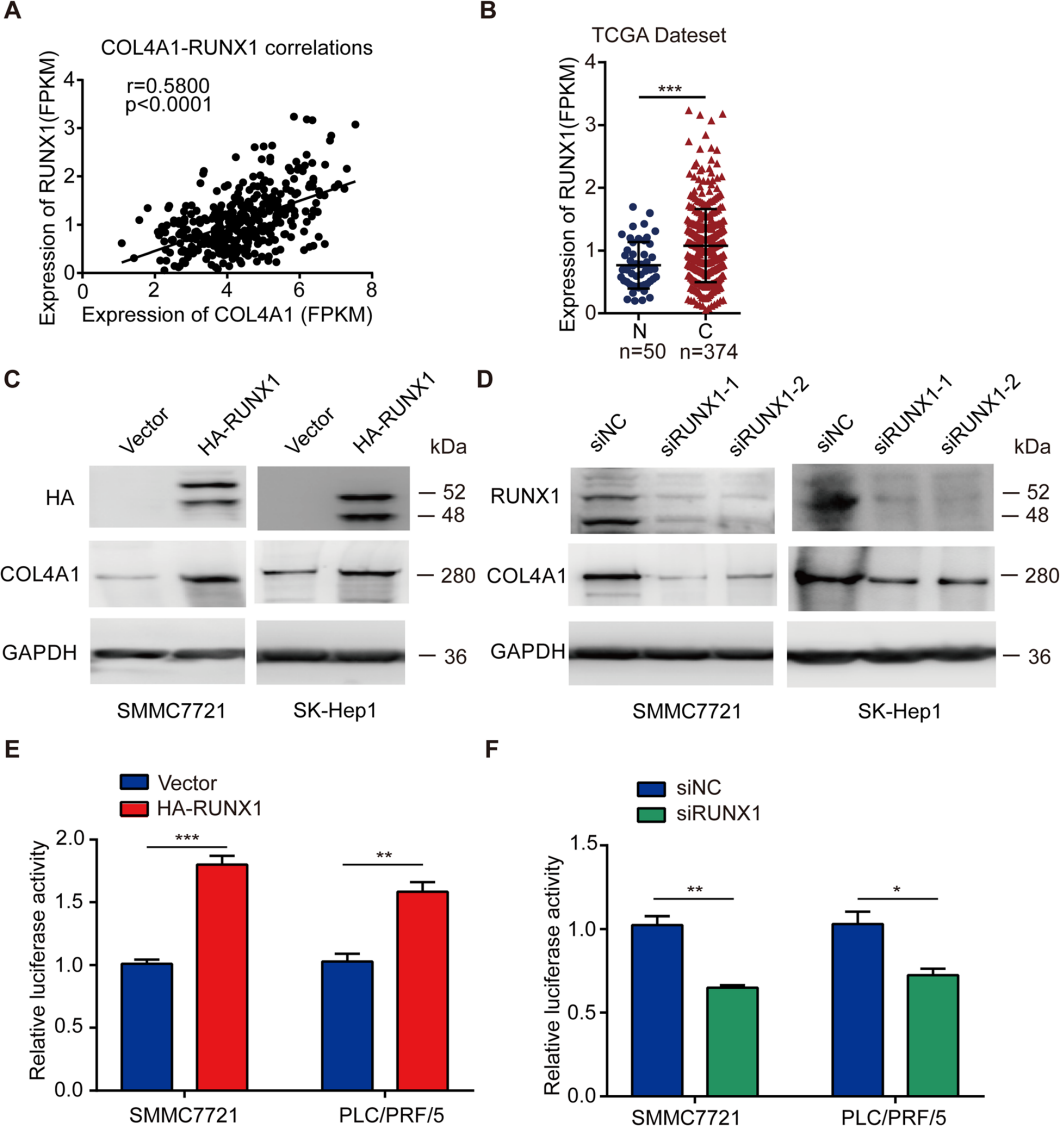
RUNX1 activates the transcription of COL4A1 in HCC**. a** Correlation between mRNA levels of COL4A1 and RUNX1. Linear regression analysis showed the positive correlation between mRNA levels of RUNX1 and COL4A1 from HCC samples in TCGA database (*r* = 0.5800, *P* < 0.0001). **b** mRNA level of RUNX1 was upregulated in cancerous tissues (C, *n* = 374) compared with noncancerous liver tissues (N, *n* = 50) from TCGA datasets. **c&d** RUNX1 regulates COL4A1 expression. Protein levels of COL4A1 and RUNX1 were detected by western blot analysis in indicated HCC cell lines after transfected with either overexpression vector (HA-RUNX1) (**c**) or si-RUNX1 (**d**). **e&f** RUNX1 activates transcription of COL4A1 by dual luciferase reporter assay. Overexpression of RUNX1 activated the transcription of COL4A1 (**e**) and knockdown of RUNX1 inhibited the transcription of COL4A1 (**f**). Student *t* test, *P < 0.05, **P < 0.01, ***P < 0.001

It has been described that RUNXs (Runt-related transcription factor family) are transcriptional factors of collagen genes [42–44]. To study whether COL4A1 is regulated by RUNXs in HCC, we screened the correlation between COL4A1 and RUNX1, RUNX2 or RUNX3 in HCC. As shown in Fig. 4a and Additional file 9: Figure S6A, the expression of COL4A1 was most positively correlated with RUNX1 (*r* = 0.5800, *P* < 0.0001). In addition, similar to COL4A1 expression, RUNX1 was highly expressed in HCC tissues, but expression levels of RUNX2 and RUNX3 were not significantly different between HCC tissues and normal liver tissues (Fig. 4b and Additional file 9: Figure S6B). We further found that overexpression of RUNX1, but not RUNX2 or RUNX3 dramatically elevated COL4A1 expression (Fig. 4c and Additional file 9: Figure S6C&D). In addition, knockdown of RUNX1 significantly decreased expression of COL4A1 in SMMC7721 cells and SK-Hep1 cells (Fig. 4d). Next, the promoter of COL4A1 was cloned into pGL3 basic vector. As shown in Fig. 4e, overexpression of RUNX1 activated the transcription of COL4A1 in SMMC7721 cells and PLC cells with dual-luciferase assay system. Knockdown of RUNX1 inhibited the transcription of COL4A1 (Fig. 4f). Several putative binding sites of RUNX1 locating in COL4A1 promoter region were found by an online website JASPAR (http://jaspar.genereg.net/) (Additional file 9: Figure S6E). We further analyzed correlation between RUNX1 expression and clinical stages of HCC. Similar with COL4A1, the expression of RUNX1 were slightly higher in high stages of HCC than that in low stages statistically (I/II vs III/IV, *P* < 0.05) (Additional file 9: Figure S6F). These data suggest that RUNX1 is a transcriptional factor of COL4A1 and probably is one of reasons for upregulation of COL4A1 in HCC.

### Overexpression of COL4A1 activates the FAK-Src signaling

It has been reported that collagen transduces signals through binding cell surface integrin or non-integrin receptors [45]. The major receptors for Col IV are integrins such as integrin α1 (ITGA1) and integrin β1 (ITGB1) [46–48]. We analyzed the expression of ITGA1 and ITGB1 in 50 paired HCC samples and normal liver tissues from TCGA dataset. Similar expression levels of ITGA1 was detected in HCC and normal liver tissues, whereas expression of ITGB1 was slightly higher in HCC samples compared with normal liver tissues (Additional file 10: Figure S7A). And protein levels of ITGA1 were similar in HCC cell lines compared with L02 cells (Additional file 10: Figure S7B). The binding of collagen to integrin led to the activation of downstream signaling pathways. FAK is one of the major substrates of integrin and it can further phosphorylate and activate downstream signaling molecules including Src and AKT [49–52]. Several studies have showed that aberrant expression of collagens are associated with phosphorylation of ERK1/2 and STAT3 as well as the expression of metalloproteinase-9 (MMP-9), β-catenin and E-cadherin [46, 53–55].

**Fig. 5:**
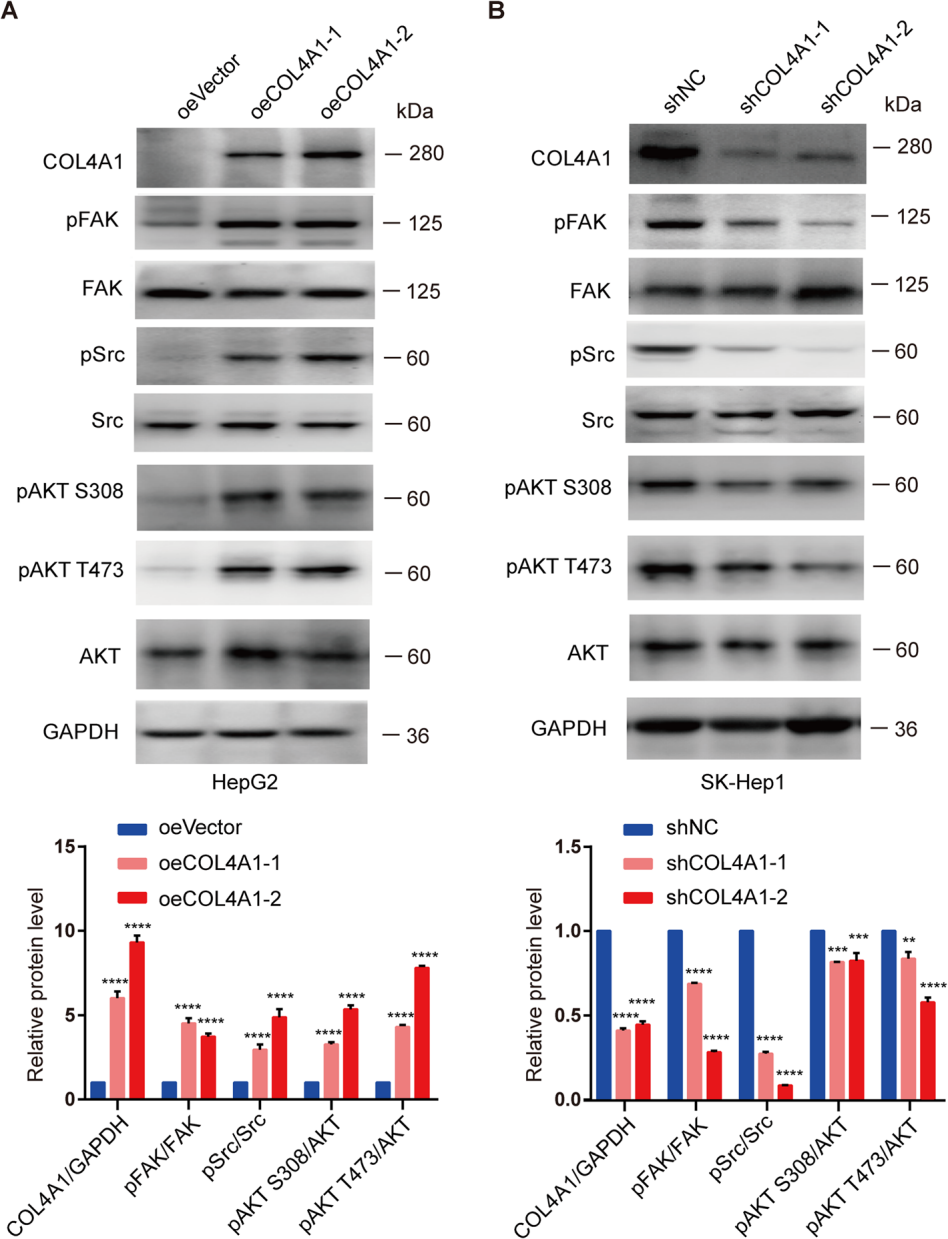
Overexpression of COL4A1 activates FAK-Src signaling**. a** Phosphorylation of FAK, Src, and AKT were analyzed by western blot in COL4A1 overexpressed HCC cells. **b** Phosphorylation of FAK, Src, and AKT were analyzed by western blot in COL4A1 knockdown HCC cells. Quantification of western blots were analyzed by Image J. Student *t* test, **P < 0.01, ***P < 0.001, ****P < 0.0001

To investigate the downstream signaling of COL4A1 in HCC, we detected the levels of phosphorylation and expression for those downstream proteins in parental cells and COL4A1 overexpression or knockdown cells. We observed that overexpression of COL4A1 increased phosphorylation levels of FAK, Src, and AKT (Fig. 5a). Knockdown of COL4A1 significantly reduced phosphorylation levels of them (Fig. 5b). Whereas, knockdown of COL4A1 had no effect on phosphorylation levels of STAT3 and ERK1/2 as well as protein levels of MMP9, β-catenin, and E-cadherin (Additional file 10: Figure S7C). Moreover, knockdown of COL4A2 decreased phosphorylation levels of FAK and Src like COL4A1 (Additional file 10: Figure S7D). However, knockdown of COL1A1 and COL3A1 had no effect on FAK activity (Additional file 10: Figure S7E&F). Together, type IV collagen promotes HCC progression by activating FAK-Src signaling.

### Inhibitors of FAK and Src selectively suppress the growth of HCC cells with high expression of COL4A1

**Fig. 6:**
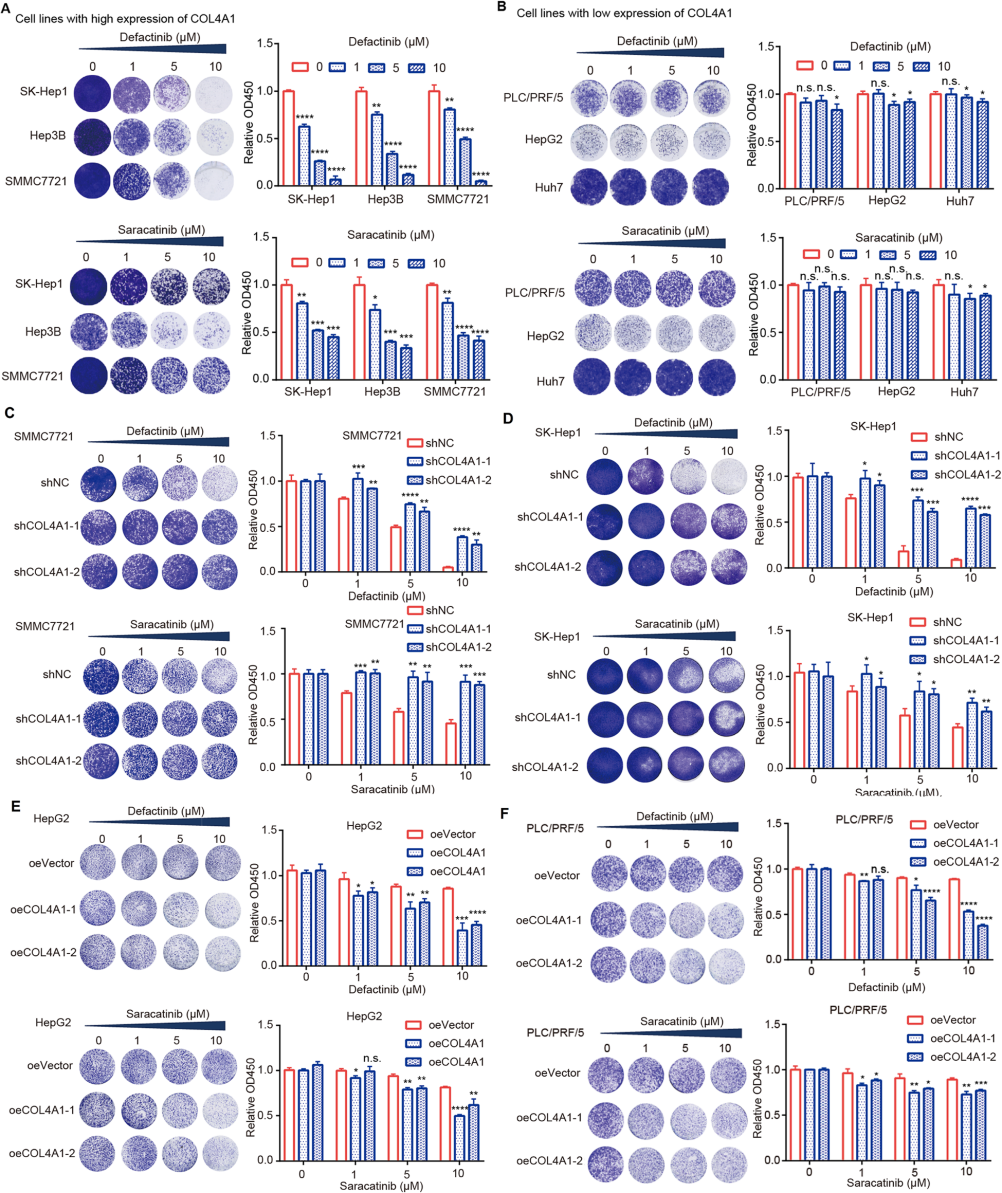
FAK or Src inhibitor selectively inhibits the cell viability of HCC cells with high expression of COL4A1. **a&b** Cell viability was tested after treatment with inhibitors. HCC cells were treated with Defactinib (FAK inhibitor) or Saracatinib (Src inhibitor) at the indicated concentrations for 48 h. HCC cells with high expression level of COL4A1 (**a**) were sensitive to Defactinib or Saracatinib treatment, but HCC cells with low expression level of COL4A1 (**b**) were resistance to Defactinib or Saracatinib treatment. **c&d** Knockdown of COL4A1 reduced the sensitive to Defactinib or Saracatinib treatment in SMMC7721 (**c**) and SK-Hep1 (**d**). Indicated cells were treated with Defactinib (FAK inhibitor) or Saracatinib (Src inhibitor) at the indicated concentrations for 48 h. **e&f** Overexpression of COL4A1 increased the sensitive to Defactinib or Saracatinib treatment in HepG2 (**e**) and PLC/PRF/5 (**f**). Indicated cells were treated with Defactinib (FAK inhibitor) or Saracatinib (Src inhibitor) at the indicated concentrations for 48 h. Cell viability was analyzed by *crystal violet staining assay (Left) and CCK8 assay (Right),* respectively. Data are presented as means ± standard deviation. Student *t* test, *P < 0.05, **P < 0.01, ***P < 0.001, ****P < 0.0001, ns, not significant

Based on promotion effects of upregulation of COL4A1 in tumor growth through activating FAK-Src signaling in HCC cells, we wonder if FAK or Src inhibitor could effectively suppress the growth of HCC cells with high expression of COL4A1. We selected Defactinib (FAK inhibitor) and Saracatinib (Src inhibitor) to evaluate their efficacy on HCC cells, because several clinical trials of these two inhibitors for cancer treatment are underway (https://clinicaltrials.gov). Results showed that both Defactinib and Saracatinib could significantly inhibit the growth of HCC cells with high expression of COL4A1 (SK-Hep1, Hep3B, and SMMC7721) (Fig. 6a), while they had little effect on inhibiting the growth of HCC cells with low expression of COL4A1 (PLC/PRF/5, HepG2, and Huh7) (Fig. 6b). To further verify whether cell sensitivity to FAK or Src inhibitors was associated with COL4A1 expression, we examined cell sensitivity to those two inhibitors after modulating COL4A1 expression in HCC cells. As expected, depletion of COL4A1 in HCC cells with high COL4A1 expression reduced their sensitive to Defactinib or Saracatinib treatment (Fig. 6c&d). Whereas, overexpressing COL4A1 in HCC cells with low COL4A1 expression could increase their sensitive to Defactinib or Saracatinib treatment (Fig. 6e&f). Therefore, COL4A1 may be a biomarker for treatment with FAK or Src inhibitor for HCC patients.

## Discussion

**Fig. 7:**
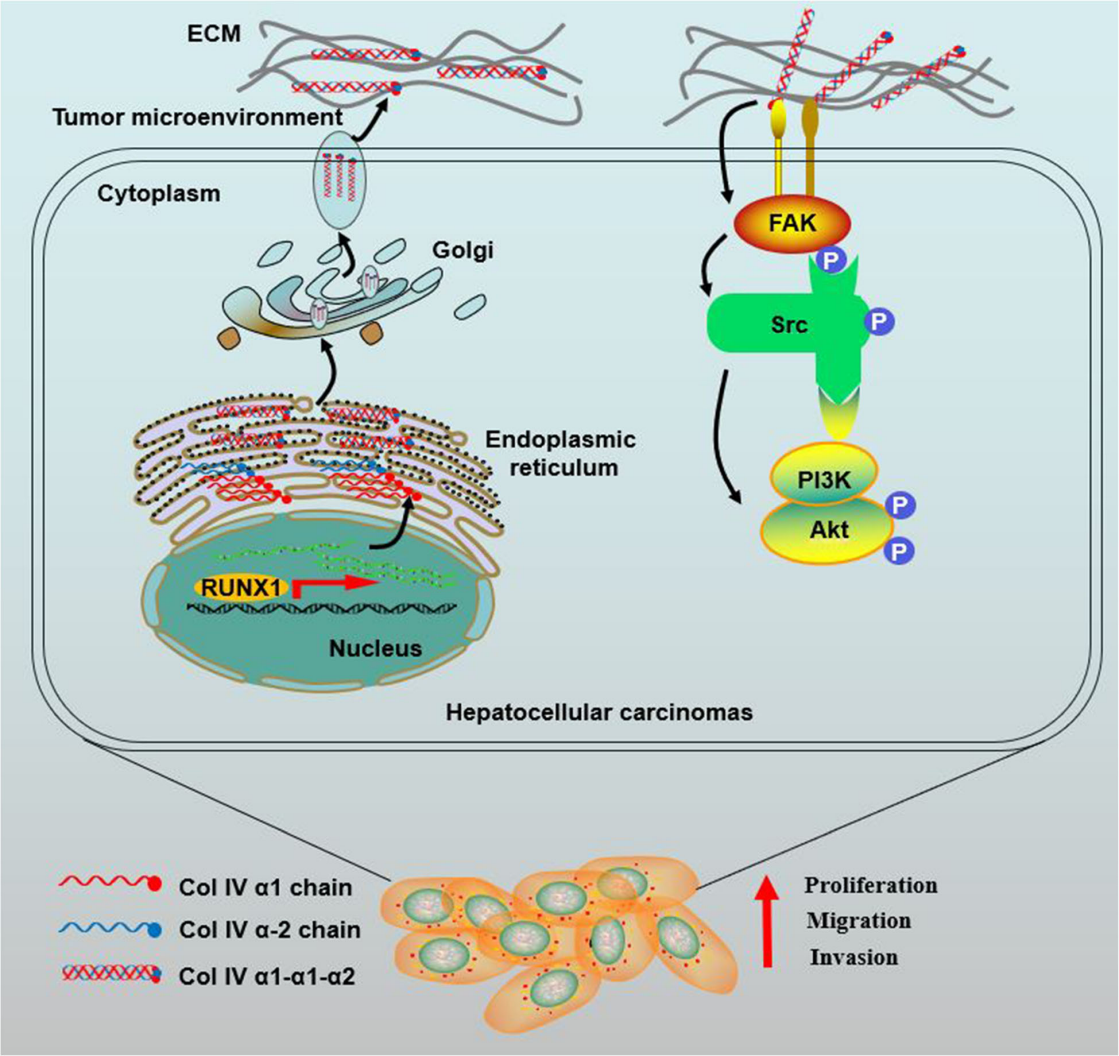
Schematic diagram of COL4A1 promoting the growth and metastasis of HCC cells. COL4A1 promotes the growth, migration and invasion of HCC cells by activating FAK-Src signaling. Col IV, Collagen type IV

In this study, we first put forward the role of COL4A1 in HCC tumorigenesis. COL4A1 is dramatically upregulated collagen gene in HCC by screening the expression patterns of all 44 collagen genes in liver cancer from the TCGA-LIHC database. COL4A1 promotes the growth and metastasis of HCC cells by activating FAK-Src signaling. RUNX1 is a transcriptional factor of COL4A1 and activates the expression of COL4A1 in HCC. Targeting FAK or Src may be an effective strategy to treat HCC patients with high expression of COL4A1 (Fig. 7).

Collagen proteins form the scaffold of tumor microenvironment and are important for tumor infiltration, angiogenesis, and metastasis [5]. Some collagen genes have been found aberrant expression during carcinogenesis in various types of cancer. However, only a few studies on the expression and function of collagen genes have been reported in HCC. Some studies reported that COL1A1 was upregulated in HCC and could promote HCC progression [17, 40, 41]. Based on bioinformatics analysis, Liu et al. reported that COL4A1 and COL4A2 were significantly correlated with hepatocarcinogenesis and HCC progression [56]. In this study, we analyzed the expression patterns of all 44 collagen genes in liver cancer from TCGA-LIHC database, and found that the expression of around 70% collagen genes are dysregulated. Among these dysregulated collagen genes, expression of COL4A1 is most abundant and significantly upregulated in HCC.

Although Col IV has been reported to associate with the progression of cancer [36, 46], the detail molecular mechanisms are not well documented. Burnier et al. showed that Col IV activated FAK in liver metastasis sites generated by different primary tumors [57]. Our data showed COL4A1 expression could affect the phosphorylation of FAK in HCC cells, suggesting that COL4A1 activates FAK signaling to promote HCC progression. Chen et al. showed that COL4A1 regulated tumor cell stiffness and migration through activation of Src and ERK1/2 [46]. Espinosa et al. reported that Col IV increased the expression and activation of ERK1/2 [53]. In breast cancer, COL4A1 induced MMP-9 expression by activating Src phosphorylation [54]. In our study, COL4A1 overexpression increased the phosphorylation of Src, but had no impact on expression level of MMP-9 and phosphorylation of ERK1/2 in HCC cells. Instead, phosphorylation of AKT was significantly regulated by COL4A1. Furthermore, COL4A2, but not COL1A1 or COL3A1 also regulated phosphorylation of FAK and Src. As FAK has been reported to affect Src and AKT activation in cancer [49–52], it is convincing that COL4A1 is involved in the proliferation and migration of HCC cells through FAK-Src signaling as type IV collagen molecules.

The reason of upregulation of COL4A1 in HCC remains unclear. In this study, we demonstrated that the upregulation of COL4A1 in HCC was due to transcriptional factor RUNX1. RUNX family, including RUNX1, RUNX2 and RUNX3, are transcription factors regulating embryonic development, proliferation, differentiation and cell growth in different tissues [58]. Three RUNXs express in different tissues and regulate different target genes through binding to different coactivators or corepressors, which lead them to regulate diverse biological processes [58, 59]. In liver cancer, our results showed that RUNX1 but not RUNX2 or RUNX3 was upregulated in tumor tissue in comparison with normal tissue. More importantly, expression level of RUNX1 positively correlates with expression level of COL4A1. Overexpression of RUNX1 but not RUNX2 or RUNX3 increased the transcription of COL4A1, and knockdown of RUNX1 reduced the transcription of COL4A1. These data suggest that RUNX1 but not RUNX2 or RUNX3 is a transcriptional factor which contributes to upregulation of COL4A1 in HCC. Some studies have reported that mRNA levels of COL4A1 are also subject to posttranscriptional control such as microRNAs [60, 61], suggesting that the expression of COL4A1 in cancer may be regulated by multiple mechanisms.

How RUNX1 is upregulated in HCC remains unknown. RUNX1 is highly active in hematopoietic cell differentiation and its overexpression is necessary for tumor formation in the skin, lungs, intestines and breasts [59, 62, 63]. In epithelial ovarian cancer, RUNX1 is significantly overexpressed due to DNA hypomethylation, and could promote cancer progression [64]. In breast cancer cells, the binding of RUNXOR lncRNA trigger CpG island DNA demethylation and activate the expression of RUNX1 [65]. Chromosomal rearrangements activate the expression of RUNX1 by perturbing its transcriptional control to contribute to acute myeloid leukemia pathogenesis [66]. Whether high expression of RUNX1 in HCC through these mechanisms is worth investigating in the future. Interestingly, COL4A1 was also upregulated in breast cancer and ovarian cancer like RUNX1 (Additional file 4: Figure S1D). It is possible that overexpression of RUNX1 in these cancer types could also activate COL4A1 expression to promote tumorigenesis.

Several studies reported that COL4A1 could be a potential therapeutic target gene in head and neck squamous cell carcinoma, colorectal carcinoma, and thyroid papillary carcinoma [36]. However, the feasibility and strategy of targeting COL4A1 for HCC patients treatment is still unclear. Considering that the oncogenic function of COL4A1 in HCC relies on FAK and Src activation, we wonder whether inhibiting FAK or Src activity is an effective approach to treat HCC patients with high expression of COL4A1. Indeed, HCC cell lines with high expression of COL4A1 such as SK-Hep1, Hep3B, and SMMC7721 were more sensitive to FAK or Src inhibitor, and knockdown of COL4A1 reduced the sensitivity of those cells. In contrast, HCC cell lines with low expression of COL4A1 such as PLC/PRF/5, HepG2, and Huh7 were more resistant to FAK or Src inhibitor, and overexpression of COL4A1 increased the sensitivity. Therefore, COL4A1 may be a potential biomarker to indicate the utilization of FAK or Src inhibitor for HCC patients treatment.

## Conclusions

In summary, our findings elucidate that COL4A1 functions as an oncogene to facilitate growth and metastasis in HCC via the activation of FAK-Src signaling. Upregulation of COL4A1 in HCC is due to transcriptional factor RUNX1. HCC cells with high COL4A1 expression are sensitive to the treatment with FAK or Src inhibitor (Fig. 7). Our study could help to better understand the mechanisms underlying HCC progression. COL4A1 maybe a biomarker and potential target for HCC therapy.

## Supplementary information

**Additional file 1: Table S1.** Antibodies used in this study.

**Additional file 2: Table S2.** Primers used in this study.

**Additional file 3: Table S3.** The list of differentially expressed collagen genes in HCC samples compared with normal liver tissues.

**Additional file 4: Figure S1.** COL4A1 is overexpressed in HCC.

**Additional file 5: Figure S2.** Knockdown of COL4A1 inhibits the proliferation, migration, and invasion in HepG2 cells and PLC/PRF/5 cells.

**Additional file 6: Figure S3.** Knockdown of COL4A2 inhibits the proliferation and migration of HCC cells.

**Additional file 7: Figure S4.** Knockdown of COL1A1 inhibits the proliferation and migration of HCC cells.

**Additional file 8: Figure S5.** Knockdown of COL3A1 has no effect on cell proliferation and migration.

**Additional file 9: Figure S6.** RUNX1 is a transcriptional factor of COL4A1.

**Additional file 10: Figure S7.** Collagen IV activates the FAK-Src signaling.

## Data Availability

The data supporting our conclusion were obtained from the TCGA database (https://cancergenome.nih.gov), Oncomine database (https://www.oncomine.org), GEO datasets (https://www.ncbi.nlm.nih.gov/gds/), and Human Protein Atlas online database (https://www.proteinatlas.org).

## Abbreviations

HCC: Hepatocellular carcinoma
TCGA-LIHC: The Cancer Genome Atlas Liver Hepatocellular Carcinoma
Col IV: Collagen type IV
COL4A1: Collagen IV alpha1 chain
COL4A2: Collagen IV alpha2 chain
COL1A1: Collagen I alpha1 chain
COL3A1: Collagen III alpha1 chain
ECM: Extra cellular matrix
shRNA-COL4A1: Small hairpin RNA expression vector targeting human COL4A1 gene
FPKM: Fragments Per Kilobase of exon model per Million mapped reads
siRNA: Small interfering RNA
SAM: CRISPR/Cas9 Synergistic Activation Mediator
RTCA: Real-time cell analyzer
qRT-PCR: Quantitative real-time polymerase chain reaction
NC: Negative control
ns: Not significant
