# Supplementary material for: COL4A1 promotes the growth and metastasis of hepatocellular carcinoma cells by activating FAK-Src signaling
Source: J Exp Clin Cancer Res. 2020 Aug 3;39:148. doi: 10.1186/s13046-020-01650-7 (PMC7398077; doi:10.1186/s13046-020-01650-7)
Supplement: Supplementary file 1 — Additional file 1: Table S1. Antibodies used in this study. [file 13046_2020_1650_MOESM1_ESM.docx]

**Additional file 1: Table S1. Antibodies used in this study**

| **Antibodies** | **Application** | **Catalog number** | Company | Clonality | **Dilution** |
| --- | --- | --- | --- | --- | --- |
| COL4A1  (Collagen IV) | WB, IHC | ab6586 | Abcam | Polyclonal | 1:1000 |
| COL4A2 | WB | 55131-1-AP | Proteintech Group | Polyclonal | 1:1000 |
| COL1A1 | WB | ab34710 | Abcam | Polyclonal | 1:1000 |
| COL3A1 | WB | ab7778 | Abcam | Polyclonal | 1:1000 |
| GAPDH | WB | sc-25778 | Santa Cruz | Polyclonal | 1:2000 |
| RUNX1 | WB | ab23980 | Abcam | Polyclonal | 1:1000 |
| HA-tag | WB | sc-805 | Santa Cruz | Polyclonal | 1:1000 |
| c-MYC | WB | sc-40 | Santa Cruz | Monoclonal | 1:1000 |
| p-Src (Tyr416) | WB | #2101 | Cell Signaling Technology | Polyclonal | 1:1000 |
| Src | WB | #2123 | Cell Signaling Technology | Monoclonal | 1:1000 |
| p-FAK (Tyr397) | WB | #8556 | Cell Signaling Technology | Monoclonal | 1:1000 |
| FAK | WB | #3285 | Cell Signaling Technology | Polyclonal | 1:1000 |
| p-AKT (Tyr308) | WB | #2965 | Cell Signaling Technology | Monoclonal | 1:1000 |
| p-AKT (Ser473) | WB | #4060 | Cell Signaling Technology | Monoclonal | 1:1000 |
| AKT | WB | #9272 | Cell Signaling Technology | Polyclonal | 1:1000 |
| p-STAT3 (Tyr705) | WB | #9145 | Cell Signaling Technology | Monoclonal | 1:1000 |
| STAT3 | WB | #9139 | Cell Signaling Technology | Monoclonal | 1:1000 |
| p-ERK1/2 (Thr202/Tyr204) | WB | #4376 | Cell Signaling Technology | Monoclonal | 1:1000 |
| ERK1/2 | WB | #4695 | Cell Signaling Technology | Monoclonal | 1:1000 |
| β-Catenin | WB | #8480 | Cell Signaling Technology | Monoclonal | 1:1000 |
| E-Cadherin | WB | #3195 | Cell Signaling Technology | Monoclonal | 1:1000 |
| MMP9 | WB | 10375-2-AP | Proteintech Group | Polyclonal | 1:1000 |
| Integrin alpha-1 | WB | 22146-1-ap | Proteintech Group | Polyclonal | 1:1000 |
