# Supplementary material for: COL4A1 promotes the growth and metastasis of hepatocellular carcinoma cells by activating FAK-Src signaling
Source: J Exp Clin Cancer Res. 2020 Aug 3;39:148. doi: 10.1186/s13046-020-01650-7 (PMC7398077; doi:10.1186/s13046-020-01650-7)
Supplement: Supplementary file 2 — Additional file 2: Table S2. Primers used in this study. [file 13046_2020_1650_MOESM2_ESM.docx]

**Additional file 2: Table S2. Primers used in this study**

| **Accession No.** | **Primer name** | **Primer sequences**  **（5’ to 3’）** | | **Product Size (bp)** | |
| --- | --- | --- | --- | --- | --- |
| **Primers for** **qRT-PCR** | | | | | |
| NM_001101 | β-actin | Forward  Reverse | TTGTTACAGGAAGTCCCTTGCC  ATGCTATCACCTCCCCTGTGTG | | 101 |
| NM_001845 | COL4A1 | Forward  Reverse | CAGGCACCCCATCTGTTGAT  CATTGCCTTGCACGTAGAGC | | 129 |
| NM_001846 | COL4A2 | Forward  Reverse | TTATGCACTGCCTAAAGAGGAGC  CCCTTAACTCCGTAGAAACCAAG | | 207 |
| NM_000088 | COL1A1 | Forward  Reverse | GGAATGAAGGGACACAGAGGTT  AGTAGCACCATCATTTCCACGA | | 78 |
| NM_000090 | COL3A1 | Forward  Reverse | GGAGCTGGCTACTTCTCGC  GGGAACATCCTCCTTCAACAG | | 198 |
| **Primers for shRNA** | | | | | |
| NM_001845 | COL4A1 shRNA-1 | Forward  Reverse | GATCCGGAGCGAGATGTTCAAGAAGCCTTCCT  GTCAGAGCTTCTTGAACATCTCGCTCCTTTTTG  AATTCAAAAAGGAGCGAGATGTTCAAGAAGCT  CTGACAGGAAGGCTTCTTGAACATCTCGCTCCG | | |
|  | COL4A1 shRNA-2 | Forward  Reverse | GATCCCAGGCTCTAAGGGTGATATCTTCCTGTC  AGAATATCACCCTTAGAGCCTGTTTTTG  AATTCAAAAACAGGCTCTAAGGGTGATATTCT  GACAGGAAGATATCACCCTTAGAGCCTGG | | |
| **Primers for sgRNA** | | | | | |
| NM_001845 | COL4A1 sgRNA-1 | Forward  Reverse | CACCGACGCGGGAGCCTCTTGAGTG  AAACCACTCAAGAGGCTCCCGCGTC | | |
|  | COL4A1 sgRNA-2 | Forward  Reverse | CACCGAGCGCGGAGCCCTGGTGTCC  AAACGGACACCAGGGCTCCGCGCTC | | |
| **Sequences for siRNA** | | | | | |
| NM_001846 | COL4A2 siRNA-1 | Forward  Reverse | CGGGUGUGAAGAAGUUUGAdTdT  UCAAACUUCUUCACACCCGdTdT | | |
|  | COL4A2 siRNA-2 | Forward  Reverse | GGCAGAAAGGUGAGCCUUAdTdT  UAAGGCUCACCUUUCUGCCdTdT | | |
| NM_000088 | COL1A1 siRNA-1 | Forward  Reverse | GGAUCUGCGUCUGCGACAAdTdT  UUGUCGCAGACGCAGAUCCdTdT | | |
|  | COL1A1 siRNA-2 | Forward  Reverse | GGCUAUGAUGAGAAAUCAAdTdT  UUGAUUUCUCAUCAUAGCCdTdT | | |
| NM_000090 | COL3A1 siRNA-1 | Forward  Reverse | CCGGUCCUAAAGGAAAUGAdTdT  UCAUUUCCUUUAGGACCGGdTdT | | |
|  | COL3A1 siRNA-2 | Forward  Reverse | GAUGCUAUCAAGGUAUUCUdTdT  AGAAUACCUUGAUAGCAUCdTdT | | |
| [NM_001754](https://www.ncbi.nlm.nih.gov/nuccore/NM_001754.5) | RUNX1 siRNA-1 | Forward  Reverse | CCAGGUUGCAAGAUUUAAUdTdT  AUUAAAUCUUGCAACCUGGdTdT | | |
|  | RUNX1 siRNA-2 | Forward  Reverse | GACAUCGGCAGAAACUAGAdTdT  UCUAGUUUCUGCCGAUGUCdTdT | | |
| **Subcloning primers** | | | | | |
| [NM_001754](https://www.ncbi.nlm.nih.gov/nuccore/NM_001754.5) | RUNX1  CDS | Forward  Reverse | CCGCTCGAGCACATCTGCTGTGCTATTAA  CCGGAATTCATGGCTTCAGACAGCATATTTG | | |
| NM_001024630 | RUNX2 CDS | Forward  Reverse | GGAAGATCTATGGCATCAAACAGCCTATTC  CCGCTCGAGTCAATATGGTCGCCAAACAG | | |
| NM_001031680 | RUNX3 CDS | Forward  Reverse | CCGGAATTCATGGCATCGAACAGCATCTTC  CCGCTCGAGTCAGTAGGGCCGCCACACGGC | | |
| NG_011544 | COL4A1  promoter | Forward  Reverse | CGGGGTACCCTGCACTTTGAGAAGCTCTG  CCGCTCGAGGAGCGCGGCGGGCCGAGCTC | | |
