# Supplementary material for: COL4A1 promotes the growth and metastasis of hepatocellular carcinoma cells by activating FAK-Src signaling
Source: J Exp Clin Cancer Res. 2020 Aug 3;39:148. doi: 10.1186/s13046-020-01650-7 (PMC7398077; doi:10.1186/s13046-020-01650-7)
Supplement: Supplementary file 3 — Additional file 3: Table S3. The list of differentially expressed collagen genes in HCC samples compared with normal liver tissues. [file 13046_2020_1650_MOESM3_ESM.docx]

**Additional file 3: Table S3. The list of differentially expressed collagen genes in HCC samples compared with normal liver tissues**

| **Upregulated collagen genes (n=27)** | COL1A1, COL1A2, COL2A1, COL4A1, COL4A2, COL4A5, COL4A6, COL5A1, COL5A2, COL5A3, COL6A3, COL7A1, COL8A1, COL9A1, COL9A2, COL9A3, COL10A1, COL11A1, COL12A1, COL13A1, COL15A1, COL16A1, COL21A1, COL22A1, COL23A1, COL24A1, COL27A1 |
| --- | --- |
| **Downregulated collagen genes (n=4)** | COL6A6, COL14A1, COL18A1, COL25A1 |
