# Supplementary figures and images for: COL4A1 promotes the growth and metastasis of hepatocellular carcinoma cells by activating FAK-Src signaling
Source: J Exp Clin Cancer Res. 2020 Aug 3;39:148. doi: 10.1186/s13046-020-01650-7 (PMC7398077; doi:10.1186/s13046-020-01650-7)

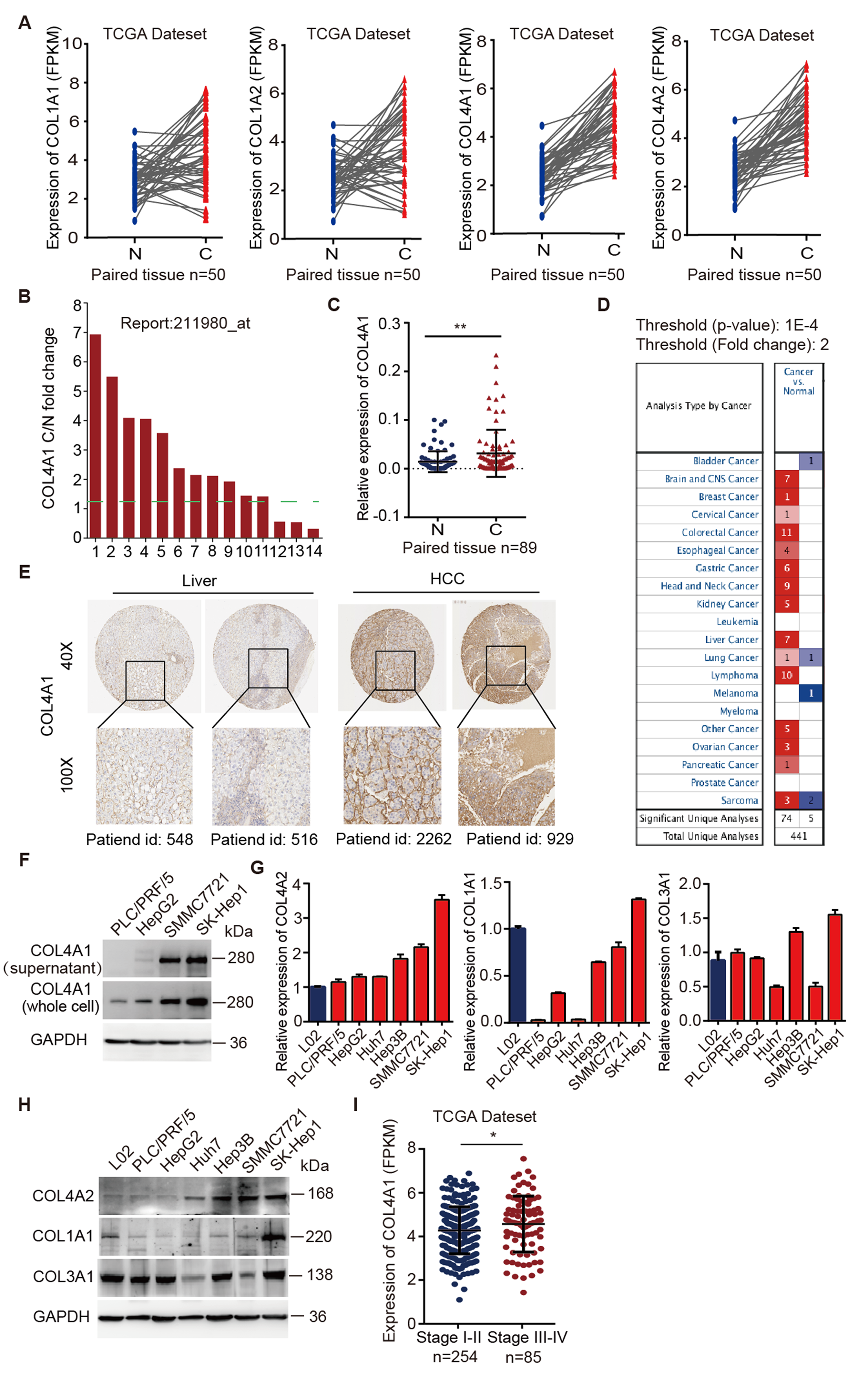

Supplement: Supplementary file 4 — Additional file 4: Figure S1. COL4A1 is overexpressed in HCC. [file 13046_2020_1650_MOESM4_ESM.tif]

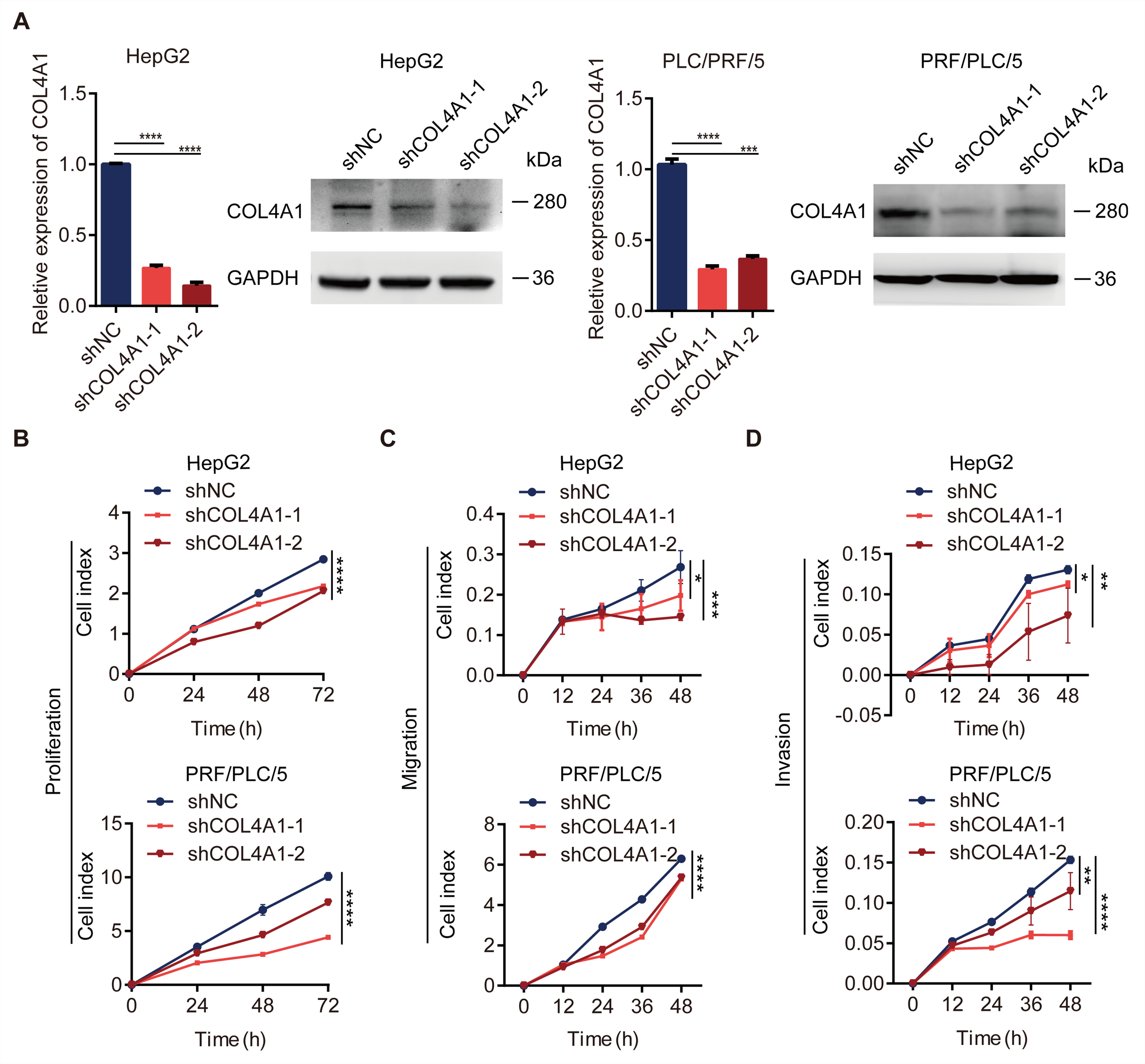

Supplement: Supplementary file 5 — Additional file 5: Figure S2. Knockdown of COL4A1 inhibits the proliferation, migration, and invasion in HepG2 cells and PLC/PRF/5 cells. [file 13046_2020_1650_MOESM5_ESM.tif]

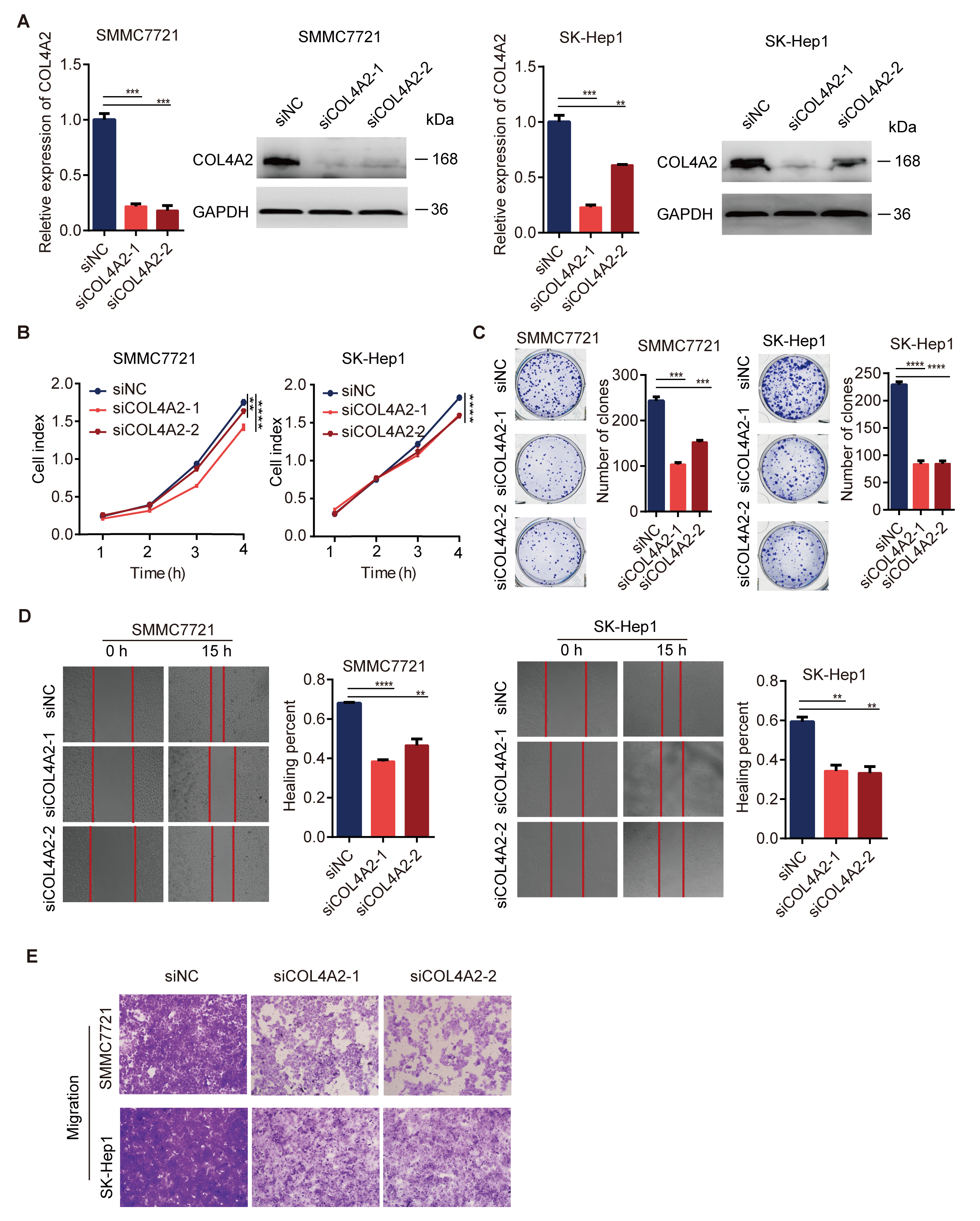

Supplement: Supplementary file 6 — Additional file 6: Figure S3. Knockdown of COL4A2 inhibits the proliferation and migration of HCC cells. [file 13046_2020_1650_MOESM6_ESM.tif]

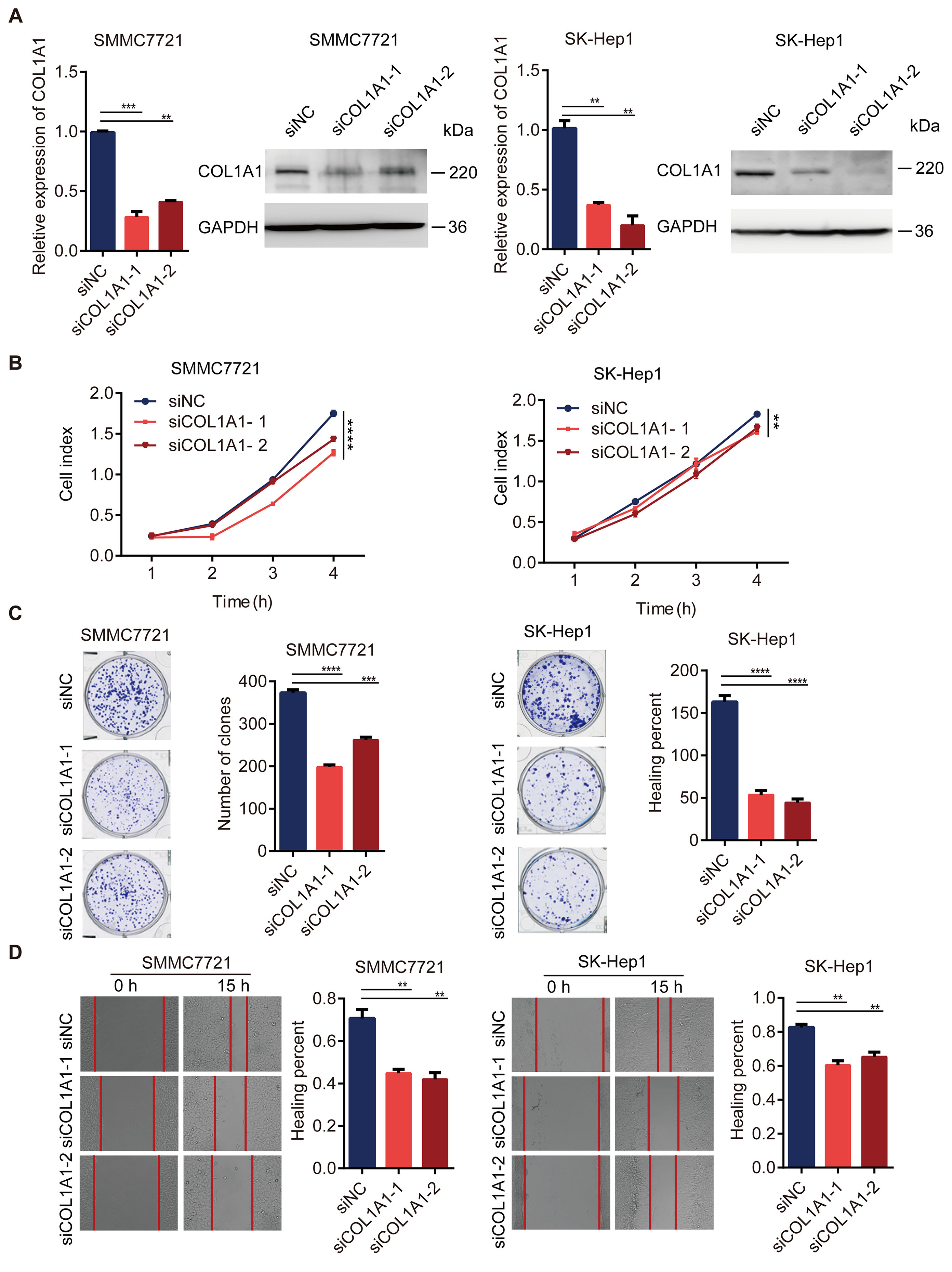

Supplement: Supplementary file 7 — Additional file 7: Figure S4. Knockdown of COL1A1 inhibits the proliferation and migration of HCC cells. [file 13046_2020_1650_MOESM7_ESM.tif]

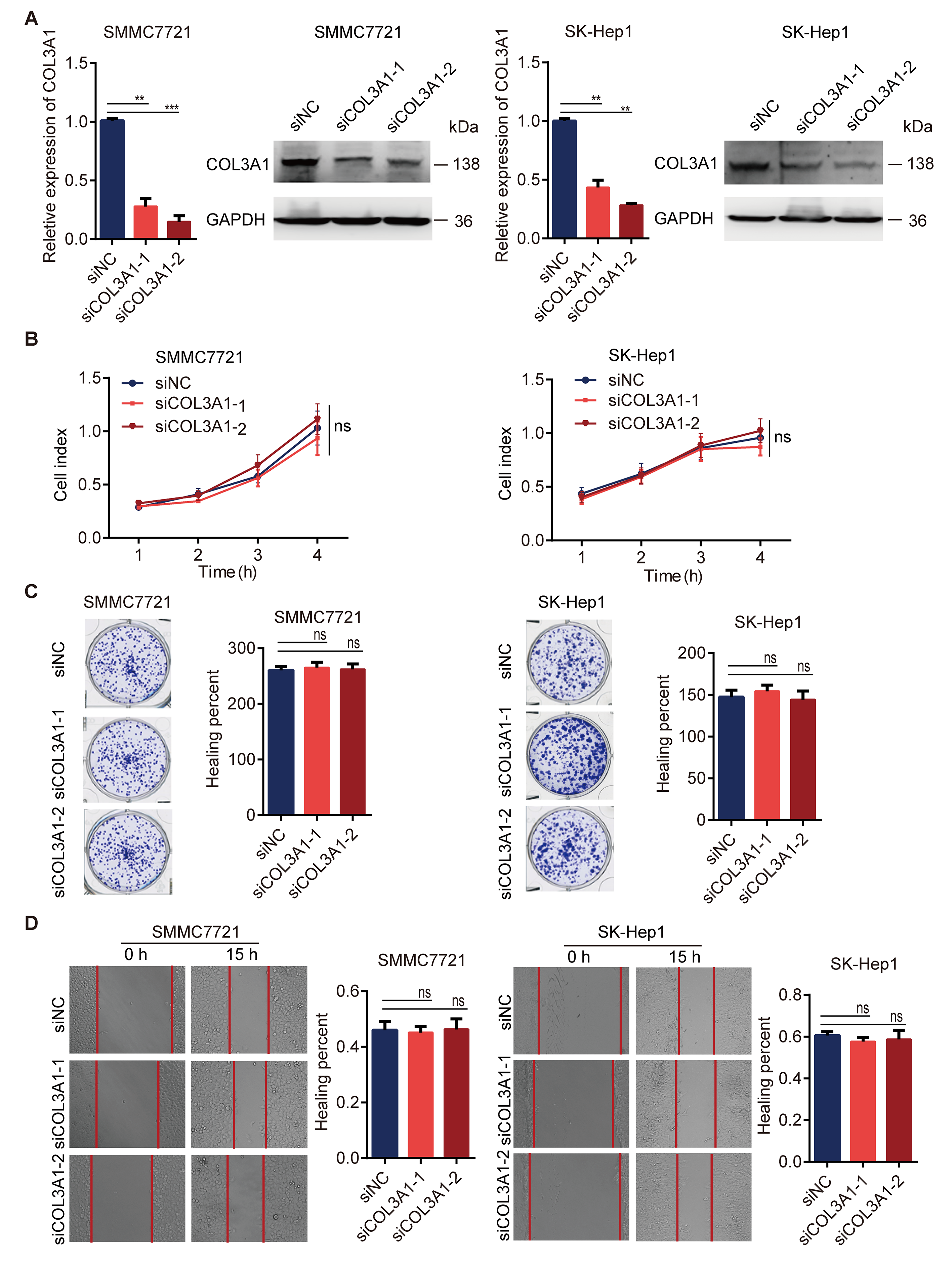

Supplement: Supplementary file 8 — Additional file 8: Figure S5. Knockdown of COL3A1 has no effect on cell proliferation and migration. [file 13046_2020_1650_MOESM8_ESM.tif]

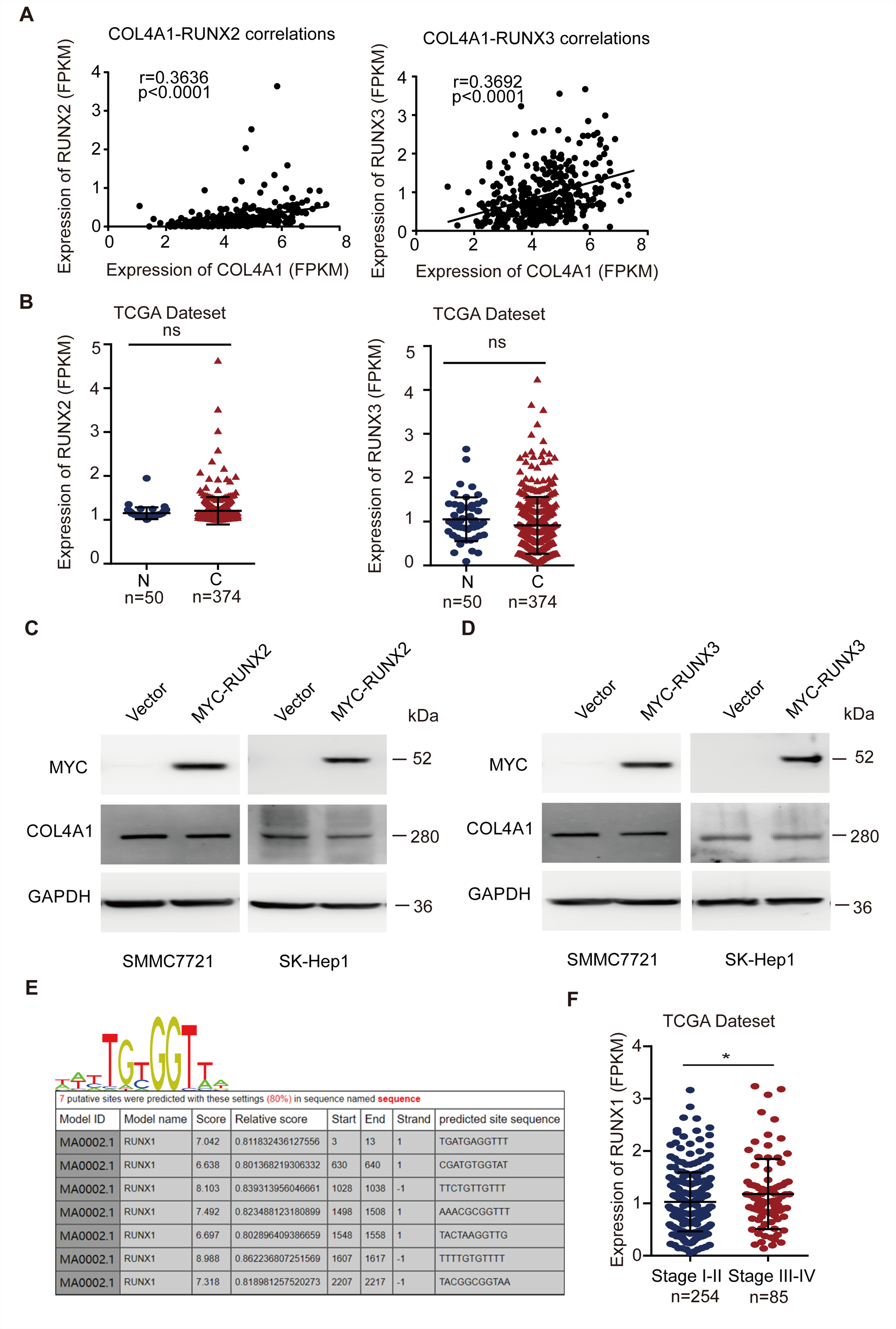

Supplement: Supplementary file 9 — Additional file 9: Figure S6. RUNX1 is a transcriptional factor of COL4A1. [file 13046_2020_1650_MOESM9_ESM.tif]

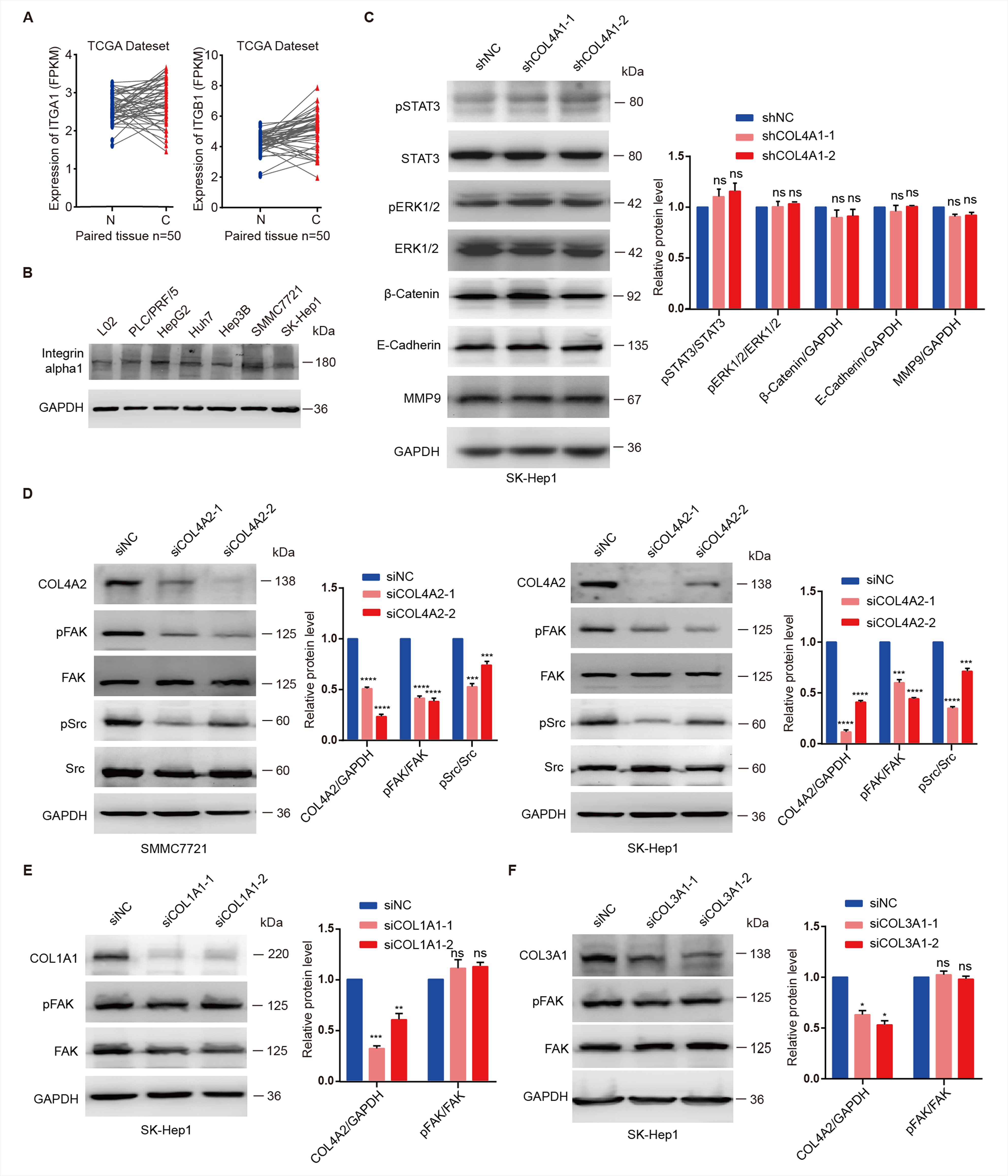

Supplement: Supplementary file 10 — Additional file 10: Figure S7. Collagen IV activates the FAK-Src signaling. [file 13046_2020_1650_MOESM10_ESM.tif]
